# Neighborhood Social Deprivation and Caregiving Intensity for Community-Dwelling Older Adults With Disabilities

**DOI:** 10.1093/geroni/igaa057.2502

**Published:** 2020-12-16

**Authors:** Chanee Fabius, John Mulcahy, Safiyyah Okoye, Jennifer Wolff

**Affiliations:** Johns Hopkins Bloomberg School of Public Health, Baltimore, Maryland, United States

## Abstract

There is growing interest in the role of “place” in the provision of long-term services and supports (LTSS) for older adults with disabilities, who receive ~16 billion hours of care per year from family and unpaid caregivers, but information is lacking. Using data from the 2015 National Health and Aging Trends Study (NHATS) linked to census-tract-level information from the American Community Survey, we described the association between caregiving intensity (hours of care received per week) and neighborhood social deprivation among N=2125 community-dwelling older adults with disabilities. Individuals receiving 40 hours or more of help per week had greater levels of functional impairment and dementia, and more often lived in neighborhoods at the highest quartile of social deprivation compared to those receiving fewer than 20 hours of care (26.8% vs. 21.7%, respectively). Findings have policy implications for targeting LTSS strategies toward addressing inequities in social determinants of health for vulnerable populations.

